# Tire–Road Contact Area on Asphalt Concrete Pavement and Its Relationship with the Skid Resistance

**DOI:** 10.3390/ma13030615

**Published:** 2020-01-30

**Authors:** Di Yun, Liqun Hu, Cheng Tang

**Affiliations:** 1 School of Highway, Chang’an University, Xi’an 710064, China; 2 Key Laboratory of Special Area Road Engineering of Ministry of Education, Chang’an University, Xi’an 710064, Shaanxi, China; 3 School of Civil Engineering and Transportation, South China University of Technology, Gunagzhou 510640, China

**Keywords:** asphalt concrete, skid resistance, texture, multi-scale roughness, real contact area, 3D-printing

## Abstract

Sufficient pavement skid resistance is one of the essential factors to ensure traffic safety. The real contact area (*A*r) between the tire and road is significant for understanding and improving the skid resistance performance. In this study, the tire–road contact area is measured by squeezing a smooth underside-dyed rubber block into the specimens, using a self-designed fixture mounted on the universal test machine. The three-dimensional (3D) printing technology is used to separate the specimens with multi-scale roughness. Surface texture on 29 AC pavements is obtained by a 3D scanner and qualified by the root-mean-square surface height (*S*q), to investigate the impact of pavement texture on the *A*r. The skid resistance on 23 AC road sections is measured using the T2GO system, and the pavement texture is recorded, to discuss the influence of the *A*r on the skid resistance. The results indicate that the multi-scale roughness rarely affects the measured contact area once the concerned wavelength is less than 0.6 mm. The *A*r decreases with the *S*q following a power function but has weak correlation with the friction coefficient. This study could provide an in-depth understanding of the tire–road contact and lays a foundation for optimizing the contact-related pavement performance.

## 1. Introduction

Vehicle crash is usually the result of a combination of factors, of which skid resistance is one. Skid resistance is a condition parameter that characterizes the contribution a road surface makes to the total level of friction available at the contact patch between a road surface and vehicle’s tire during acceleration, braking, and cornering maneuvers [1]. A sufficient level of skid resistance allows the vehicle to be controlled under different driving conditions. Additionally, with the increase in the skid resistance, its effect as a factor in crashes is also reduced.

Skid resistance consists of four components, namely the adhesion, hysteresis, viscous damping, and cohesion [2]. Under different test conditions, the contribution from the four components to friction varies. For example, adhesion is dominant at low slip speeds, while the hysteresis and viscous damping could significantly affect the friction at high slip speeds and wet conditions [3]. Some researchers suggested that the adhesion, hysteresis, and cohesion are related to the real contact area (*A*r) between the tire and the surface of road [4]. Fwa et al. reported that *A*r has a significant effect on the measured friction [5]. Therefore, understanding the friction between a rubber block and a hard and rough substrate requires a deep insight into the contact formation of the two solids.

The roughness on asphalt pavement is represented at many scales. Pavement texture was defined as a combination of a microtexture with a wavelength range of 0–0.5 mm, a macrotexture with a wavelength range of 0.5–50 mm, a mega-texture with a wavelength range of 50–500 mm, and unevenness with a wavelength range of 500 mm–50 m. The microtexture is produced by the surface properties of individual chippings or other particles of the surface. The macrotexture is always obtained by using suitable proportions of aggregate and motor of the mix or by surface-finishing techniques [6]. It is thought the microtexture and macrotexture dominate the tire–road friction [7,8]. In the mid-20th century, Bowden and Tabor proposed the importance of surface roughness, and opened a new era in the study of rough surface contacts [9].

Previous studies indicated that differences in the surface roughness may result in different results about the *A*r [10,11,12].Unlike the studied shapes, such as a parabola, sphere, triangle, and sine, the pavement texture has its intrinsic characteristics, e.g., surface skewness caused by the compaction process and accumulated vehicle loads. Therefore, the contact between the tire and the pavement needs further research. The multi-scale roughness of pavement could result in a gradual decrease in the contact area with the scale, at which the contact area is observed (observing scale). This means that, from the macro-perspective, the tire-pavement contact seems to occur on the whole contact patch, while from the micro-perspective, the contact patch will be ‘separated’ by the asperities on the pavement, so that the contact only occurs on several spots. Cesbron et al. used the digital pressure sensing devices to capture the real contact area between the tire and specimens made up of asphalt mixture [13]. However, the roughness involved by the equipment is limited since low resolution cannot identify the real contact area on each asperity in detail, especially when the surface consists of small protruding of aggregates. The results showed that there was no significant relationship between the contact area and the friction coefficient obtained by the British pendulum tester. The observing scale may affect the obtained contact area and its relationship with the performance of pavement. Persson’s contact mechanism showed that when the observing scale is small enough, its effect on the contact area would be weakened. For the tire–road contact area, there may be a suitable scale, in which the value of *A*r can be obtained without much effort [14].

## 2. Aims and Scopes

**Figure 1:**
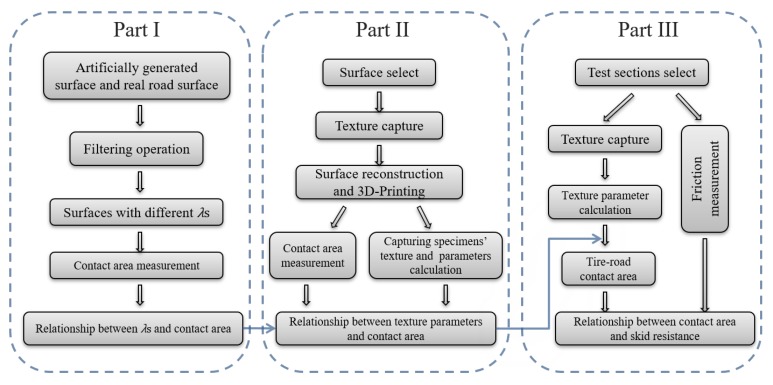
Experimental procedure of this study.

The tire–road contact area is related to pavement skid resistance. This study intends to investigate the effect of observing scale on the observed tire–road contact area and try to obtain the area of real contact (*A*r). Then, the relationship between the *A*r and the texture on asphalt concrete pavement is discussed. Based on this, the contact area between the test device and the pavement can be predicted, and the influence of contact area on the skid resistance is discussed. The flow chart representing the scope and aim of this study is shown in Figure 1.The study provides an in-depth understanding of the pavement performance related to tire–road contact and lays a foundation for establishing the relationship between the performance and pavement texture.

## 3. Experimental

Pavement texture, tire pressure, and tread rubber hardness were essential factors affecting the contact area. Other factors included contact time, temperature, operation (acceleration, deceleration, and turning), and tire age. All these factors will affect the tire–road contact, so it is hard to consider the real conditions. This study simplified the real contact situation by squeezing the planar rubber into a 3D printed pavement under static uniform pressure at the temperature of 20 °C in a laboratory.

This study set the load as 50, 500, 1000, 1500, and 2000 N, which represented the pressure values of 0.018, 0.18, 0.35, 0.53, and 0.71 MPa, respectively. The pressure beneath the commonly-used friction tester (e.g., Dynamic Friction Tester, Wehner/Schulze machine) and the vehicle tire was involved. Besides, the hardness of the used rubber was HSA55 and HSA68, which corresponded to the hardness of rubber on the British Pendulum tester, dynamic friction tester, and tire tread, respectively.

### 3.1. Specimens Preparation

#### 3.1.1. Specimens for Part I

The contact occurs at asperities between a rubber and a pavement surface, which has roughness at a wide range. When observing the contact area at a smaller scale, the finer roughness could be seen, and the observed contact area decreases. The effect of observing scale on the contact area would be weakened significantly when the scale is small enough. Thus, the observed area will not be infinitely small, and a real contact area can be detected by not too much effort. Investigating the effect of observing scales on the contact area helps in obtaining the real contact area.

In reality, there is a specific contact area between a rubber and a particular pavement surface. This study simulated the surface seen at different scale by changing the involved shortest wavelength, which can be obtained by a low-pass filter. When keeping the longest wavelengths (λ_l_) on the surface of specimens as a constant, the shorter smallest wavelength (λ_s_) meant finer roughness was involved, as what was seen at a smaller scale.

**Figure 2:**
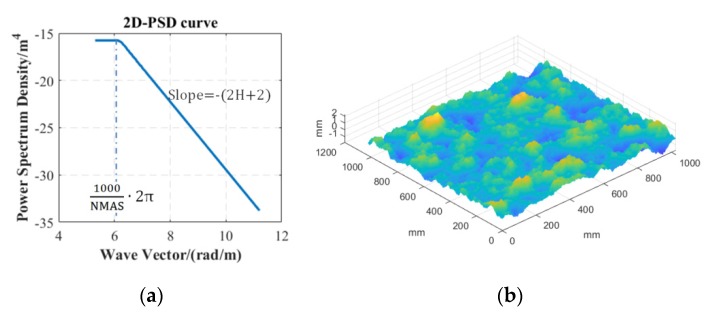

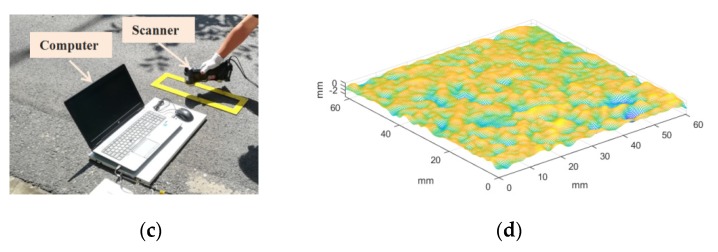
(**a**) PSD curve used for generating (**b**) an artificial surface, and (**c**) reconstructed AC pavement surface captured by (**d**) the 3D scanner.

The concerned surfaces in the present study were based on 1) artificially generated surface from Power Spectrum Density (PSD) curve; and 2) asphalt concrete pavement. Figure 2a shows the PSD curve used for generating the surface, on which the fractal dimension (H), the root-mean-square surface height (*S*q), and the nominal maximum aggregate size (NMAS) of the asphalt mixture are the essential factors for determining the curve shape. Based on the investigation of these determining factors in the field, the *S*q, which equals to the area behind the PSD curve, was set to be 0.5 mm, the H was set to be 0.8, the nominal maximum aggregate size was 12.6 mm. The generated surface is with self-affine feature and Gaussian distribution, as shown in Figure 2b. The road surface was captured using a high-precision (0.1 mm horizontal resolution and 0.04 mm vertical resolution) 3D scanner (Figure 2c) (HandySCAN 3D 300^TM^, Creaform, Canada), as shown in Figure 2d.

The value of the smallest λ_s_ involved was related to the precision of 3D printer and 3D scanner. The precision of the used industrial 3D printer in this study was 0.1 mm, while the sampling interval for acquiring the texture using a 3D scanner was 0.1 mm. Therefore, the value of smallest λ_s_ obtained was about 0.2 mm according to the Shannon’s sampling theorem. The printed surfaces (60 × 60 mm^2^) can represent the obtained roughness of the surfaces.

#### 3.1.2. Specimens for Part II

Involving as many surfaces with different pavement texture as possible was necessary to investigate the effect of texture on the tire–road contact area. This study selected pavement with different mean texture depth (MTD) using the sand patch method. The mixture type in these concerned pavements is asphalt concrete, which is common in Xi’an, China. The MTD of concerned surfaces lied within the range of 0.31–1.41 mm, as shown in Table 1. The 3D models of the surfaces were reconstructed based on the point cloud, which was obtained using a 3D scanner. Then, the specimens were printed using a 3D printer. Specimens used for Part I were also included to diversify the pavement texture since the filtered surfaces can be regarded as surfaces that lose fine roughness due to polishing.

#### 3.1.3. Friction Test Sections

The 23 test sections with almost the same wear level and different texture were selected in Xi’an, China. The used mixture type in these sections was asphalt concrete. The aggregate used in the test sections was limestone.

### 3.2. Measurement of the Contact Area

**Figure 3:**
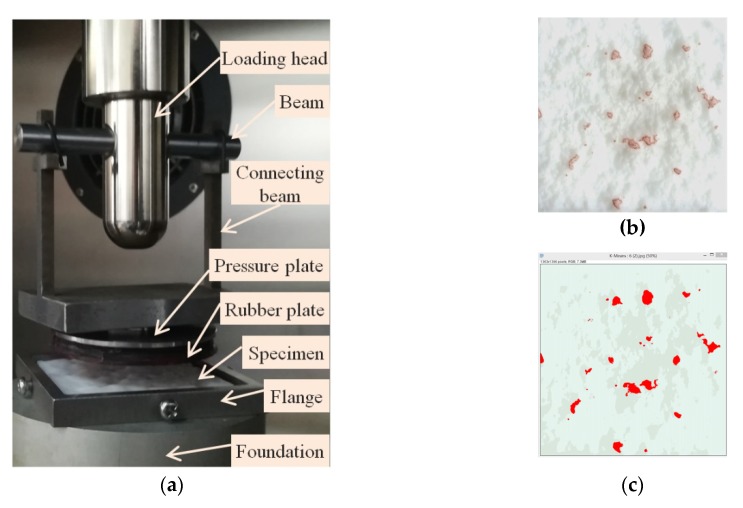
Measurement of the contact area. (**a**) self-designed fixture (**b**) the dyed area on the specimens’ surface, (**c**) the identified contact area.

The tire–road contact was simplified by squeezing a smooth rubber block dyed underside into the printed specimens. The specimens generated by the 3D-printing technology were made by the photocurable resin. They can provide a modulus of more than 3000 MPa at 20 °C (the concerned temperature when measuring the contact area), which is at the same order as the modulus of asphalt pavement. By contrast, the modulus of rubber at 20 °C is less than 3–5 MPa so that the deformations of the printed surfaces can be ignored. Once the contact occurred, the stain at the bottom of the rubber block remained on the specimen’s surface. The dye chosen in this study had a strong dyeing force, the color was in sharp contrast with the specimens’ color. More importantly, the stain could be applied thinly, so the dyed area had a high resolution to identify the fine roughness. A self-designed fixture, with a flat steel plate at the bottom, can uniformly distribute the load, was mounted on the universal test machine (UTM), as shown in Figure 3a. A specimen can be fixed with a flange plate (80 × 80 mm^2^) at the base that was correctly aligned with the loading head.

The UTM machine worked according to the set testing protocol. In this study, once the pressure reached the set value and remained stable for five seconds, the press would unload automatically. The dyed area on the specimens’ surface (Figure 3b) was recorded and measured (Figure 3c). The ratio of the measured area to the nominal contact area (area of the rubber bottom) indicated the relative contact area.

### 3.3. Measurement of the Skid Resistance

**Figure 4:**
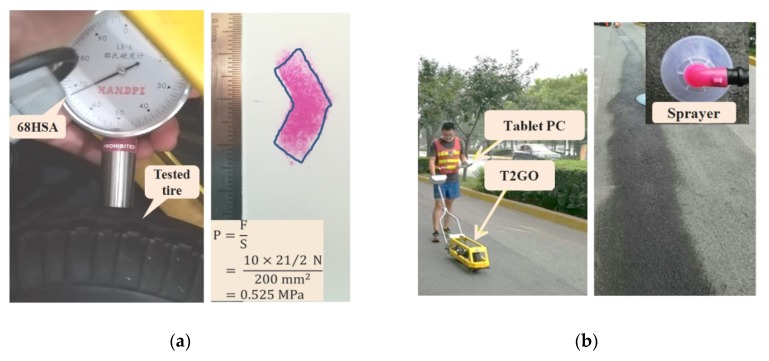
(**a**) Rubber stiffness and contact pressure measurement; (**b**) skid resistance measurement.

This study used the T2GO system (SARSYS-ASFT, Kopingebro, Sweden) to measure the friction coefficient continuously. The equipment provided a testing speed of less than 4 km/h, the minimum data-capturing interval was about 30 mm. The hardness of the tire tread rubber of the device was HSA68, the pressure was calculated to be 0.53 MPa according to the weight of the instrument (21 kg) and the contact patch recorded using the 4LW Prescale film (Figure 4a). The measurement was conducted at a stable speed of 2–3 km/h for around 10–15 m on dry pavement, followed by on the wetted pavement, where pavement wetting was achieved using a sprinkler head in front of the measuring device. The wetness was such that the water filled parts of the valleys in the texture (Figure 4b).

### 3.4. Texture Characterization

This study used standard deviation of the surface height (*S*q) to explore the relationship between the pavement texture and the contact area. The *S*q is one of the most commonly used parameters in characterizing the pavement texture, and has advantages of being less affected by the sampling interval and the size of the evaluation area. Furthermore, the *S*q has a strong correlation with other texture parameters. It can be calculated using Equation (1) [15].

(1) $$Sq=\frac{1}{A}\underset{A}{\iint }z^{2}\left(x,y\right)dxdy$$

where, *z*(*x*, *y*) is the surface height measured from the average plane with *z* = 0 within a definition area *A*. When studying the relationship between the pavement texture and the contact area, the *S*q was calculated based on the surface data of the 3D printed specimens. When predicting the contact area between the test device and the test sections for the skid resistance, the *S*q was calculated using the data of the test sections. During the capturing of the pavement texture, three positions were randomly selected within each test section. The mean value of *S*q of the three surfaces was used as the representative value of the test section.

## 4. Results

### 4.1. Contact Area and Observing Scale

**Figure 5:**
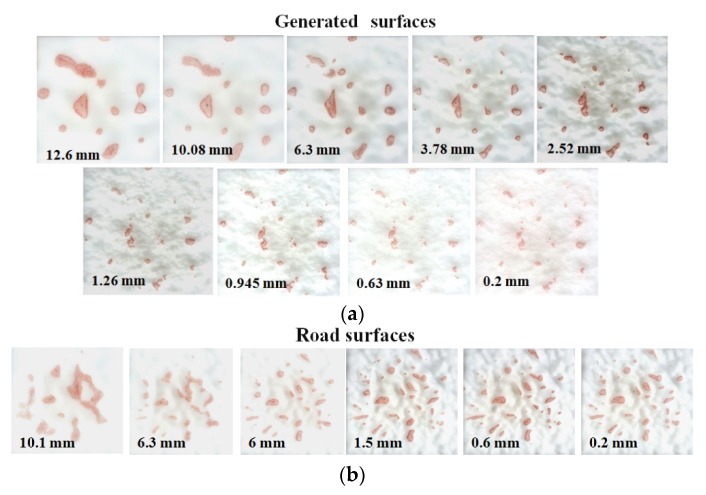
Observed contact area on (**a**) artificially generated surfaces and (**b**) AC pavement surfaces.

Figure 5 shows the effect of the observing scale on the contact area under the load of 50 N and rubber hardness of HSA55. The decrease in the minimum wavelength contained in the surface represents the fact that the contact area is observed on a reduced scale. It is clear that the contact area (red spot) rapidly thinned down or even is separated by the surface asperities when the surface involves a shorter wavelength. The effect of the contained minimum wavelength on the contact area becomes smaller, and it seems that the contact area remains constant.

**Figure 6:**
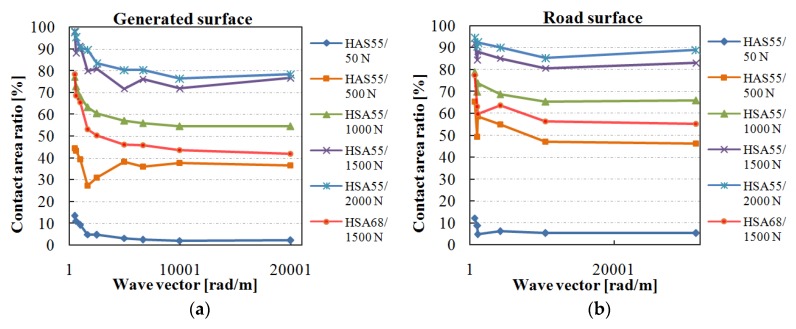
Effect of the observing scale on the contact area between a smooth rubber and (**a**) artificially generated surfaces; (**b**) road surfaces.

Figure 6 shows the effect of observing scale on the contact area between the smooth rubber and the concerned two groups of surfaces under different load (50, 500, 1000, 1500, and 2000 N) and rubber hardness (HSA55 and HSA68) conditions. The *x*-axis indicates the wave vector, which is the number of the waves per meter. It can be calculated using 2π/λ_s_, the λ_s_ refers to the smallest wavelength involved in the roughness on a specimen. The larger wave vector means a rougher surface and a smaller observing scale as illustrated in 3.1.1.

The contact area decreases dramatically with the decrease in the observing scale. Additionally, the decrease rate also reduces sharply. Therefore, the real contact area reaches a stable value for a small enough observing scale. For the group of generated surfaces, which with self-affine feature and Gaussian distribution, the wavelength corresponding to the stable contact area is about 0.63 mm. For the concerned road surface, the wavelength is about 0.6 mm.

When the contact area remains stable with the observing scale, the corresponding wavelength maybe affected by the method used for measuring the contact area. Specifically, the fine texture could be filled with the dye due to the squeezing force. Due to this reason, the surface roughness cannot separate the dyed area though the involved wavelength is short enough. For the artificially generated surface, on which there are more fine asperities, the observed contact spot still could be separated by the asperities with a wavelength of 0.6 mm. Therefore, it is inferred that the method can get the contact area at the scale of about 0.6 mm. Additionally, the ratio of decrease in the contact area drops with the decrease in the observing scale, and the ratio is pretty low (see Figure 6) before the observing scale reaches 0.6 mm. This means that the contact area between the surface and the rubber block will not decrease significantly with the observing scale though the surface consists of wavelength shorter than 0.6 mm. Therefore, the observed contact area can be regarded as the real contact area.

**Figure 7:**
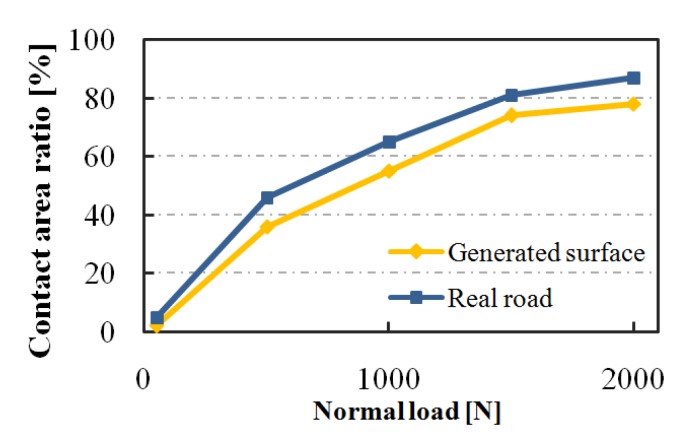
Load and the contact area.

For the group of generated surfaces, when the load is 50, 500, 1000, 1500, and 2000 N, the contact area remains stable at about 2, 36, 55, 74, and 78% of the nominal contact area (the bottom area of the smooth rubber block), respectively. For the road surfaces, the contact area becomes stable at about 5, 46, 65, 81, and 87% of the nominal contact area. This means that the real contact area increases with the increase in load, while the rate of increase slows down with the increase in load, as shown in Figure 7. Besides, when the rubber hardness is HSA55 and HSA68, and the load is 1500 N, the contact area remains stable at 74%, and 43% for the generated surface, and at 81% and 55% for the road surface, respectively. These results indicate that the harder rubber will significantly reduce the contact area.

### 4.2. Contact Area and Pavement Texture

#### 4.2.1. Under Different Loads

**Figure 8:**
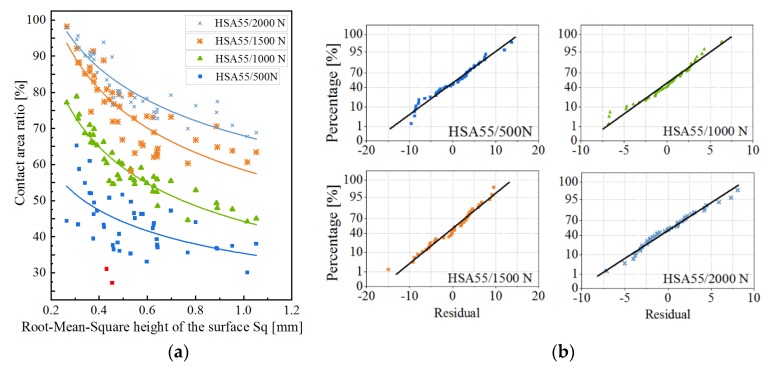
(**a**) Relationship between the contact area and the value of *S*q under different loads; (**b**) Normal probability graphs of the residual of the corresponding fitting curves.

The scatter diagram describes the relationship between the pavement texture parameter *S*q and the contact area ratio under different load forces (Figure 8). The contact area concerned in this study is at the scale of 0.6 mm. It is assumed that the contact area ratio can reach 100% when the value of *S*q approaches zero, and is close to a specific value when the value of *S*q is large. Therefore, the relationship between the contact area ratio and *S*q could be a power function. Figure 8a shows the fitting curves as the solid lines. Several points in the data series resulted in unexpected fitting, which was not consistent with the previous analysis. Thus, the abnormal values, indicated by the red color, were removed before fitting. Table 2 presents the detailed information of the curves, as well as the results of the T-test and F-test. The results show that the models and all the involved parameters are of statistical significance. On the Normal Probability Graph of residual shown in Figure 8b, the residual follows the normal distribution, which means that the model can correctly extract the factors affecting the contact area.

In reality, the value of *S*q neither reaches zero nor increases indefinitely. Instead, it lies within the range of 0.25–1.2 mm in this study. When the value of *S*q is less than 0.6 mm, the contact area decreases dramatically with the increase in *S*q, and the contact area drops by about 60%. After that, the contact area decreases slowly with the increase in the value of *S*q. Specifically, the contact area reduces by 20% for 0.6–0.8 mm of *S*q, by about 10% for 0.8–1.0 mm and 1.0–1.2 mm of the *S*q.

Generally, the higher load corresponds to a larger contact area, while the gradient of the contact area varies with the loading conditions. When the value of *S*q is less than 0.65 mm, the impact of load on the contact area is similar to that described in Section 4.1. This means that the rate of increase of contact area decreases with the increase in load. However, when the value of *S*q is large, the rate of increase does not reduce significantly with the increase in load.

#### 4.2.2. Under Different Rubber Stiffnesses

**Figure 9:**
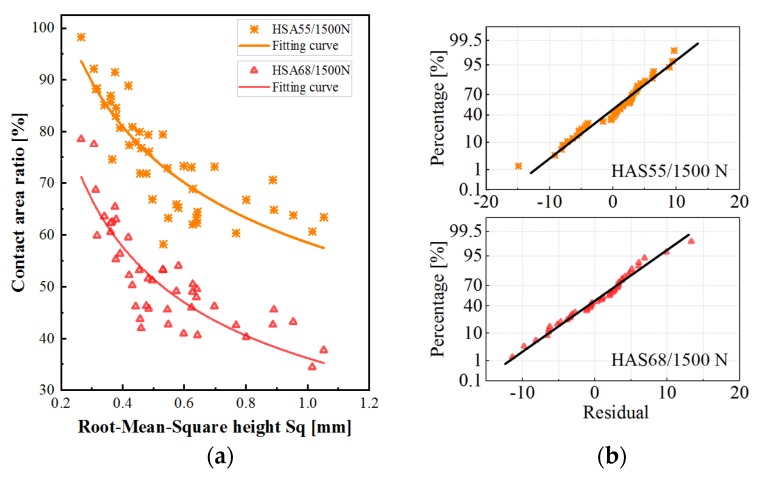
(**a**) Relationship between the contact area and the value of Sq under different rubber hardnesses; (**b**) Normal probability graphs of the residual of the corresponding fitting curves.

Figure 9 shows the relationship between the contact area and the texture parameters under different rubber hardness. From the scatter diagram, the fitting curves, the residual distribution diagram, and the detailed information provided in Table 3, it can be concluded that, within the rubber hardness involved in this study, the relationship between the contact area and the parameter *S*q follows a power function.

The softer rubber contributes to a larger contact area since the softer rubber is more prone to deformation. When the value of *S*q is less than 0.6 mm, the contact area decreases dramatically with the increase in the value of *S*q. The contact area drops by about 60%, when the value of *S*q increases from 0.6 to 0.8 mm. The contact area decreases by 10%, when the contact area increases from 0.8 to 1.0 mm and from 1.0 to 1.2 mm. Additionally, the rate of decrease of contact area under different rubber hardness is different. When the value of *S*q is less than about 0.6 mm, the harder rubber increases the rate of decrease. When the value of *S*q is larger, the rubber hardness hardly affects the rate of decrease. In this case, the distance between the curves gradually increases with the increase in the value of *S*q.

### 4.3. Skid Resistance Performance

**Figure 10:**
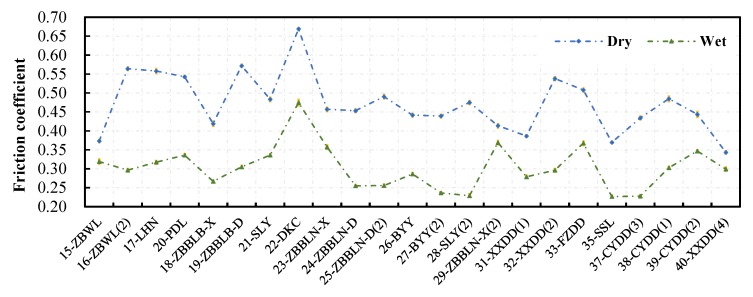
Friction coefficient measured under dry and wet conditions.

The friction coefficient of the concerned test sections measured by the T2GO device under dry and wet conditions is shown in Figure 10. The values are the average in test sections, and the 95% confidence intervals of the values are represented by the short orange lines. The confidence interval means that there is a 95% chance for the range to include the true value of the friction coefficient of the pavement. The length of the interval is very short compared to the value, so that the mean value of the friction coefficient can reasonably represent the skid resistance performance of the test section. The recorded temperature on pavement surface during the measurement is 35.8 ± 5.8 °C for the dry condition, and 34.6 ± 5.5 °C for the wet condition. Previous study suggested that the effect of temperature on the measured friction by the T2GO device was related to the surface properties of the pavement. Additionally, the increment in temperature increased the friction coefficient by maximum 0.003 for 1 °C [16]. That is, the temperature difference during the measurement could cause the difference in friction coefficient by maximum 0.03 (less than 1% of the measured friction). Thus, this study ignored the effect of temperature on the measured friction.

Generally, the measured friction coefficient under dry condition is higher than that under wet condition. The variations of the friction coefficient with the surfaces under the two conditions are similar. This means that the surface providing lower dry skid resistance has lower wet skid resistance. However, the difference in wet-dry friction varies from surface to surface, which might be due to different contact areas, or other relevant information within the contact area.

### 4.4. Contact Area Prediction

**Figure 11:**
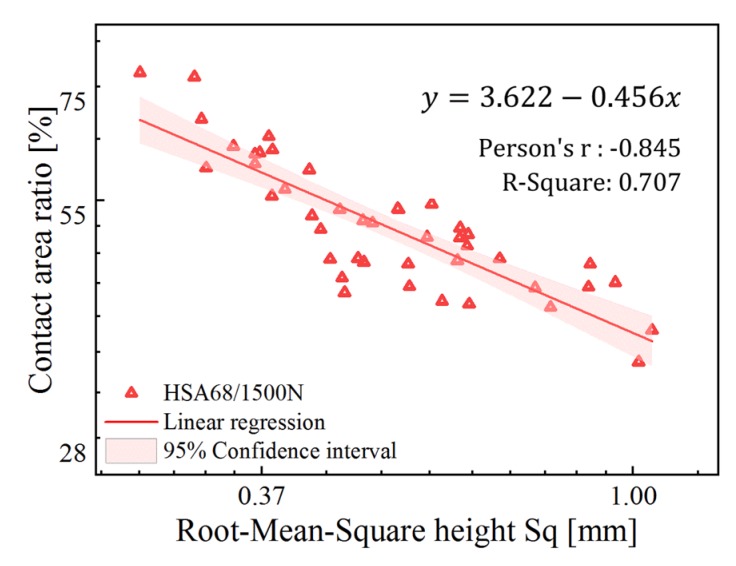
Log plot of the relationship between the contact area and the pavement texture under static condition of HSA68/1500 N.

Figure 11 shows the prediction model of the contact area under HSA68/1500 N. Both the horizontal and vertical coordinates are based on natural logarithm. The T-test and F-test indicate that the parameters and the model itself are with statistical significance. The region in light red shows the 95% confidence interval of the prediction model. The Pearson’s correlation coefficient is 0.845, and the goodness-of-fit statistic R^2^ is 0.707, indicates that the change in Sq can explain 70.7% change in the contact area.

The tire tread hardness of the test device used for measuring the friction coefficient was HSA68, and the contact pressure between the tire and road was about 0.53 MPa (corresponds to the pressure under 1500 N load). Though the viscoelastic properties of rubber are strongly temperature-dependent, the effect of temperature increment during the measurement can be negligible due to heat diffusion at low sliding speed and the narrow tire width. Therefore, the relationship between the contact area and the pavement texture under static condition of HSA68/1500 N can be used to predict the contact area between the test vehicle and the pavement.

**Figure 12:**
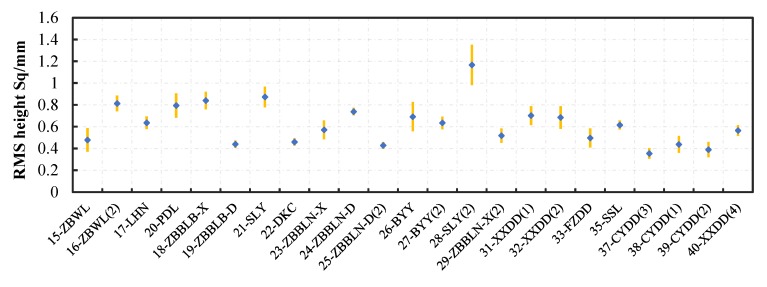
Parameter *S*q of the pavement in different test sections.

This study recorded the point cloud of three different positions in each test section. Three evaluation areas in each position were used for calculating the value of Sq. The average of value of the *S*q represents the roughness on the pavement, and the short orange lines show the standard deviation of *S*q values (Figure 12). The difference between different test sections in *S*q indicates that the concerned texture of the pavement is diverse. Additionally, the *S*q values of the test sections are not beyond the range of *S*q concerned in the prediction model. Due to this reason, the predicted values of the contact area between the test device and the pavement are reliable.

### 4.5. Contact Area and Skid Resistance

**Figure 13:**
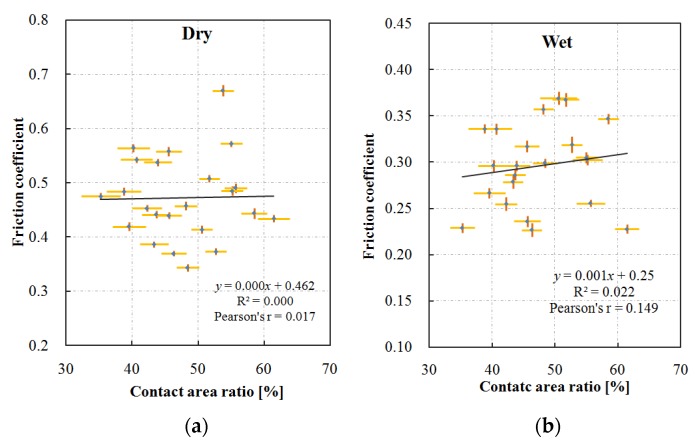
Effect of contact area on friction coefficient measured under (**a**) dry and (**b**) wet conditions.

Figure 13 shows the relationship between the contact area and the friction coefficient measured at a low-speed. The error bars show the 95% confidence interval of the variables. The dry friction coefficient fluctuates around a specific value (about 0.47). The fitting curve and the correlation coefficient show that there is no significant relationship between these two variables. In contrast, the friction coefficient measured under wet conditions increases slightly with the increase in contact area, and the fitting curve and the correlation coefficient show that there is a weakly positive correlation between these two variations.

**Figure 14:**
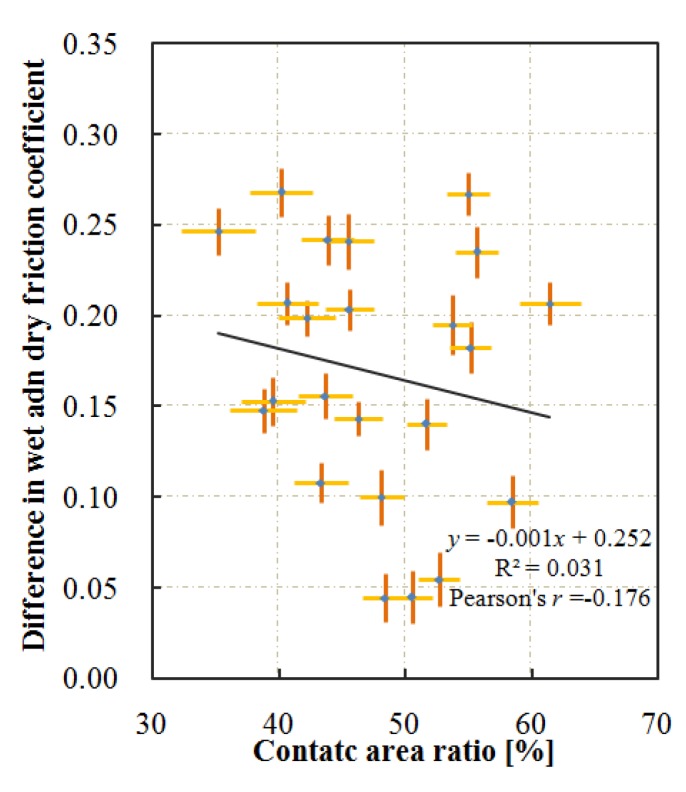
Effect of contact area on difference in friction coefficient.

In Section 4.3, the difference between the dry and wet friction coefficients varied much in different test sections, and it was speculated to be caused by the contact area. The relationship between this difference and the contact area shows that the contact area slightly reduces the difference between the dry and wet friction coefficients (Figure 14).

## 5. Discussion

This study verified that the observed contact area would change with the observing scale. It proposed 0.6 mm as the appropriate scale for the real contact area since the area tends to remain stable with the observing scale though the involved wavelength becomes shorter (Figure 6). At the small enough wavelength, the high local stresses at the rubber surface corresponds to the plastic deformation. Persson’s study assumed that, at the minimum scale, the plastic deformation occurs at 50% of the real contact area [14]. Based on a surface with the self-affine feature and Gaussian distribution, the scale was calculated as greater than 10 μm [17]. Kennedy et al. suggested that the scale is not determined by the plastic deformation of the rubber, even on clean and dry pavements. This was due to the reason that the rubber debris from the rolling tire affected the tire–road contact [18]. The particle size between the contact pairs determines the minimum wavelength contributing to the contact, and the size of tire wear debris is generally 1–100 μm [19]. By contrast, the observing scale suggested in this study is much larger than that proposed in the theoretical studies. Tires can only contact the top part of the pavement. The accumulated traffic smoothens the top part of the pavement surface, especially when the limestone is used. Therefore, on the pavement with a smoother top part than on a surface with self-affine feature and Gaussian distribution, there could not be such a small wavelength to affect the contact area.

The contact area has been found to increase proportionally with the load, based on the surfaces with self-affine features or surfaces with spherical asperities with a Gaussian distribution of heights [14]. This study drew a similar conclusion when the pavement surface has larger *S*q value (>0.6 mm). However, when the surfaces have smaller *S*q, the rate of increase of contact area decreases. The difference is likely to be caused by the flatter ‘plateau’ in the top part of the pavement, with which the tire rubber come into contact. The contact area between the rubber and surfaces with small *S*q is larger than that with larger *S*q. Therefore, the rubber can hardly overcome the resistance from ‘plateau’ to embedded texture. When the contact area reaches around 80%, the contact area increases more slowly (Figure 8).

This study drew a similar conclusion that the increase in the value of *S*q leads to a decrease in the contact area, as reported in some previous studies [14]. In contrast, the rate of decrease in the contact area decreases rather than remaining constant as some previous studies have suggested. This could be due to the increase in the value of *S*q, which makes rubber more prone to deformation. Due to this reason, the rubber contacts more area of the substrate. It turns out that the speed of contact area reduction with the value of *S*q decreases (Figure 7).

There is no significant difference in dry tire–road friction, though the contact area varies significantly (Figure 13a). Several previous studies drew the same conclusions. For example, Persson suggested that the range of the surface wavelength which contribute to the friction changes from one surface to another. On smooth surfaces, the range of wavelength contributing to the friction coefficient became wider [19]. According to the theory of rubber friction, developed by Persson, the dry friction on different surfaces would not differ much. Besides, Ahmad investigated the dry friction of surfaces with different MPD using British pendulum number (BPN) and found that there was no significant difference between the BPN values measured under dry conditions [20]. The same authors found that the high friction treated surfaces (HFST), which had significantly larger MTD, resulted in a significantly dry BPN. However, the large MTD might cause energy loss of rubber block of the pendulum and result in a much higher, but unreliable BPN value [21]. Therefore, this study suggests that the contact area has no significant effect on the low-speed friction measured under dry conditions.

There is a negative correlation between the contact area and the value of *S*q (Figure 8 and Figure 9). Furthermore, the contact area has a weakly positive correlation with the wet friction coefficient (Figure 13b). This means that the low-speed wet friction coefficient between the tire and the surface with larger *S*q would be lower. It seems to be contrary to the expectation, in which the wet friction coefficient increases with the value of *S*q of the pavement. The main reason for the positive effect of *S*q on friction coefficient results from higher drainage capacity. The better drainage capacity is essential when testing the wet friction coefficient at high sliding speeds (>60 km/h) since high speed and a thick film of water on the pavement surface encourage a vehicle to aquaplane [22]. However, the test speed in this study is quite low (<10 km/h), and the water occupied only the bottom of the texture. Besides, the size of the contact patch is small, so the difference in the drainage has negligible impact on the friction coefficient, and the different relationship between *S*q and the friction coefficient is reasonable. Water that cannot escape from the texture would affect the friction coefficient [10]. In this study, the water would be trapped in the microtexture. Therefore, the pavement texture is smoothened, and the frictional coefficient decreased. On the surfaces with higher *S*q, the rubber deformed more easily, the dry friction coefficient is more likely to depend on the microtexture on the top asperities. Therefore, on the surfaces with higher *S*q, the wet friction coefficient drops more than that under dry conditions (Figure 14). This means that the larger rubber-pavement contact area causes a higher low-speed wet friction coefficient.

This study ignored the effect of temperature difference during the measurement on the friction based on the previous research, which suggested a positive influence of temperature on the friction measured by T2GO device [16]. However, the typical test equipment—e.g., British Pendulum Tester and locked-wheel trailer—suggested a negative influence of temperature on friction. Although variations in the tire inflation pressure and the viscoelastic properties of the rubber may explain the temperature related observations, more detailed experiments are needed to gain an understanding of the underlying phenomena. Adjusting the measured friction coefficient to a reference temperature could make the results more comparable and more reliable.

Generally, the low-speed friction measurement device used in this study can evaluate the skid resistance of the city roads, where the vehicle drive at less than 50 km/h. The conflict, in which the relationship between the pavement roughness and the friction coefficient under dry and wet conditions is different, could suggest a further study on an appropriate range of the pavement roughness for the high skid resistance pavement. Besides, the friction behavior between the sliding rubber and the test surface cannot be fully described by the contact area, other factors within the contact area may need to be investigated.

## 6. Conclusions and Future Prospects

The real contact area of tire-pavement is crucial in understanding the formation of friction between the two solids and other contact-related pavement performance. This study simplified the tire–road contact by squeezing a rubber into the concerned substrate, investigated the contact area when it was observed at different scales, studied the relationship between the pavement texture and the contact area, and then, discussed the effect of contact area on the friction coefficient. Based upon the results, following conclusions are drawn.

1. The contact area between the rubber and the pavement decreases sharply with the scale when it is observed, the ratio of the decrease also drops with the scale. Before the observing scale decreases to approximately 0.6 mm, the contact area begins to stabilize. The contact area obtained at the scale of 0.6 mm can be regarded as the real contact area.
2. The real contact area is significantly affected by the roughness of the pavement surface, and decreases with *S*q following a power function. Taking the reduction of the contact area within the studied values of *S*q, the contact area reduces by about 60% when *S*q increases from 0.25 mm to 0.6 mm, decreases by about 20% when Sq increases from 0.6 mm to 0.8 mm, reduces by 10% when *S*q increases from 0.8 to 1.0 mm and from 1.0 to 1.2 mm.
3. The load and rubber hardness would not change the effect of pavement texture on the real contact area, though higher pressure and softer rubber would cause a larger contact area between the rubber and the pavement. On surfaces with low *S*q (<0.6 mm), the rate of increase of contact area decreases with the increase in load, while on surfaces with higher *S*q, the growth rate of contact area remains almost unchanged. Additionally, when the rubber becomes softer, the contact area decreases less with the value of *S*q on surfaces with low *S*q.
4. The low-speed tire–road friction coefficient under dry conditions is unaffected by the real contact area, while the friction coefficient under wet conditions is weakly positively related with the real contact area.

The surface texture of the substrate in this study is in accordance with that on the asphalt concrete pavement, and the rubber hardness and load values can cover the practical conditions for the vehicle and the commonly-used friction coefficient testing methods. Therefore, this study would help understand the effect of multi-scale pavement texture on the contact area and provide an in-depth understanding on the relationship between the contact area and the friction coefficient. However, the studied static contact may be quite different from the dynamic contact due to the effect of temperature accumulation and frequency excitation on rubber, especially when the tire–road contact is at high speed. Therefore, the dynamic tire–road contact should be considered in future. Additionally, other types of the pavement need to be concerned and other factors within the contact area need to be investigated to understand the contact more clearly. The effect of the temperature on the friction should be considered to make the result more comparable and more reliable.

## Figures and Tables

**Table 1:** Mean texture depth (MTD) of the selected surfaces

No.	Surface	MTD	No.	Surface	MTD	No.	Surface	MTD	No.	Surface	MTD
1	FZDD-1	0.31	9	ZBWL-1	0.49	17	WYL1	0.631	25	WYL06	0.864
2	FZDD-3	0.342	10	XX3-3	0.509	18	ZBBLD-2	0.658	26	WYL03	0.875
3	CY1-3	0.403	11	xx2-3	0.53	19	ZBBLD-3	0.689	27	SLY-2-3	0.93
4	SS1-3	0.421	12	WYL7	0.56	20	SLY-1-2	0.722	28	SLY-2-2	1.101
5	FZDD-2	0.47	13	WYL5	0.584	21	DKC-3	0.757	29	SLY-2-1	1.414
6	SS1-2	0.471	14	LHN-2	0.6	22	ZB8B-X-2	0.757	-	-	-
7	xx2-2	0.489	15	LHN-1	0.62	23	YCL03	0.766	-	-	-
8	XX3-1	0.49	16	WYL3	0.628	24	YCL08	0.771	-	-	-

**Table 2:** Parameters of the fitting curves for different loads

Test Condition	Eq.	a		b		P (F-Test)
		Value	P (T-Test)	Value	P (T-Test)	
HSA55/500N	*y* = *a* × *x^b^*	34.579	0.00	−0.359	0.00	0.00
HSA55/1000N		44.262	0.00	−0.417	0.00	0.00
HSA55/1500N		58.510	0.00	−0.354	0.00	0.00
HSA55/2000N		67.968	0.00	−0.266	0.00	0.00

**Table 3:** Parameters of the fitting curves for different rubber hardness

Test Condition	Eq.	a		b		P (F-Test)
		Value	P (T-Test)	Value	P (T-Test)	
HSA55/1500N	*y* = *a* × *x^b^*	58.510	0.00	−0.354	0.00	0.00
HSA68/1500N		36.197	0.00	−0.509	0.00	0.00
